# Canadian after-school care providers’ perceived role promoting healthy lifestyles: a focused ethnography

**DOI:** 10.1186/s12889-020-09369-y

**Published:** 2020-08-25

**Authors:** Pierrette H. Elias, Genevieve Montemurro, Lauren Sulz, Brian Torrance, Kate E. Storey

**Affiliations:** 1 School of Public Health, University of Alberta, 3-50 University Terrace, 8303-112 Street, Edmonton, AB T6G 2T4 Canada; 2 Department of Secondary Education, Faculty of Education, University of Alberta, 350 Education Centre South, 11210 - 87 Ave, Edmonton, Alberta T6G 2G5 Canada; 3 Ever Active Schools, 11759 Groat Road, Edmonton, Alberta T5M 3K6 Canada

**Keywords:** Youth health, After-school, Care provider, Health promotion

## Abstract

**Background:**

After-school care programs have garnered interest in recent years as the hours of 3:00–6:00 p.m. are an opportune time for children to engage in healthy behaviours, specifically healthy eating and physical activity. Care providers are major influencers within the after-school care setting, impacting health promoting opportunities for children. However, little is known regarding the role care providers play in health promotion interventions in the after-school care setting, specifically those using comprehensive approaches. The purpose of this research was to explore care providers’ role and experience promoting healthy eating and physical activity through the after-school care health promotion intervention School’s Out … Let’s Move (SOLMo). SOLMo was guided by the evidence-based comprehensive school health framework. SOLMo had two main goals: [1] to serve a healthy snack with vegetable or fruit, and milk or water as the drink; [2] to include 30 min of moderate to vigorous physical activity. The intervention included resources and coaching for care providers to promote healthy eating and physical activity for children and took place in four after-school sites over a six-month period. Three of four sites were located in a school. The primary researcher was engaged with the sites over 22-months.

**Methods:**

This research was guided by the qualitative method focused ethnography. Semi-structured interviews with care providers (*n* = 13) taking part in SOLMo were conducted. Participant observation was included as part of data generation to further understand care provider roles. Latent content analysis was utilized iteratively and concurrently throughout data generation.

**Results:**

Overall, care providers were supportive of promoting health behaviours in the after-school setting. Through analysis, five themes and eight subthemes emerged related to care providers’ role and experience promoting healthy eating and physical activity through SOLMo: 1) enhanced awareness; 2) improved programming; 3) strong relationships; 4) collaborative approach; and 5) role tension.

**Conclusions:**

As major influencers, care providers play a crucial role in promoting healthy lifestyle behaviours for children. This research provides valuable insight into this role and the implementation of comprehensive health promotion approaches in the after-school setting. Findings contribute to the implementation knowledge base and help inform the promotion of healthy lifestyle behaviours for children.

## Background

Overweight and obesity in children continues to be a public health concern [1]. Recent data show the prevalence of childhood obesity has stabilized this past decade in Canada [2]. However, with nearly one-third (31%) of children aged 5–11 years with overweight or obesity, this prevalence remains high [3, 4]. Consequences of childhood obesity are well-recognized and include chronic illnesses, poor mental health, and reduced quality of life. Thus, effective strategies to promote healthy lifestyles for children remain a priority.

The etiology of childhood obesity is complex, requiring a multifaceted approach to understand the numerous determinants [1, 4]. Lifestyle factors, such as unhealthy diets and sedentary behaviours, are known determinants of unhealthy weights [4, 5], and it is known that Canadian children are not meeting national guidelines for optimal health [6–8]. Schools, with their ability to reach the vast majority of children, are an ideal setting for health promotion [5]. As well, it has been shown that healthy students learn better [9]. Programs within schools, specifically those taking a comprehensive school health (CSH) approach, have proven to be effective in improving healthy eating (HE), physical activity (PA), and weight status of children. CSH is an internationally recognized, evidence-based health promotion approach for improving children’s health and well-being while promoting academic achievement [10, 11]. CSH and its’ equivalencies (e.g., Health Promoting Schools and the Whole School, Whole Community, Whole Child Model) are increasingly being adopted globally as a best-practice approach to build healthy school communities. As a holistic approach, CSH is comprised of four distinct, but interrelated components: (1) social and physical environment; (2) teaching and learning; (3) policy; and (4) partnerships and services [12]. As such, the guiding principles of CSH are ideal for other community settings, such as after-school care (ASC).

The after-school hours of 3:00–6:00 p.m. have garnered interest in recent years among organizations, groups, and individuals working to improve the health and wellbeing of children and youth (13). Those working to support the creation of health promoting environments during school hours, have identified the after-school time period as an important window for intervention [13, 14]. Specifically, these critical hours provide opportunities to promote health behaviours such as improving HE and PA [15–20]. It has been recognized that these non-school hours may provide an added benefit to conventional school-day strategies, which have proven effective (10) but face unique barriers to implementation. Challenges to school-based programs include lack of time, and pressure to improve academic performance (19). After-school programs, in contrast, also reach large numbers of children and youth (20) and can flexibly provide both structured and unstructured health promotion activities to students, through a program-based (i.e., non-curricular) model. While health promotion interventions in the ASC setting have demonstrated improvements in health behaviours [18], few interventions have used comprehensive approaches such as CSH. Dobson, Scott and Naylor [21] implemented a comprehensive approach to promoting healthy lifestyle behaviours within the ASC setting. Specifically, this study (*Healthy After School*) used the CSH framework to promote HE and PA within participating ASC programs in the province of British Columbia. The primary goal was to help school-aged children maintain or achieve healthy weight trajectories by supporting physical activity and healthy eating and providing healthy choices within the after-school care setting. Findings showed improvements in PA levels among children, and more healthful choices offered by sites (i.e., fruits, vegetables, and whole grains). Dobson and colleagues also reported on the vital role of ASC providers, stating that staff were essential for the success of the intervention. This finding was also supported by qualitative implementation research related to the Healthy After School guidelines, which identified that a high level of staff skill and commitment was needed to organize and facilitate PA across sites [22]. While ASC providers are regarded as individuals who have an impactful role in the ASC setting, little is known regarding their role in promoting healthy behaviours, specifically for interventions taking a CSH approach. In this study, the after-school health promotion intervention, School’s Out...Let’s Move (SOLMo), provided an opportunity to explore the role of the care provider.

### Intervention: School’s out … Let’s move

School’s Out … Let’s Move (SOLMo) was an after-school intervention, developed by Ever Active Schools [23], which aimed to improve HE and PA opportunities for children. Ever Active Schools is a provincial initiative in Alberta, Canada, designed to create and support healthy school communities, using a CSH approach. SOLMo was guided by the CSH approach, and had two main goals: (1) to serve a healthy snack with vegetable or fruit, and milk or water as the drink; (2) to include 30 min of moderate to vigorous PA (MVPA) by encouraging movement development and promoting the love of movement. Research was an important component of SOLMo, and used to assess the implementation and effectiveness of the intervention. To measure effectiveness, SOLMo used a quasi-experimental pre-post evaluation design and included child (measured body mass index, piezoelectric time-stamped pedometers, direct snack observations) and site-level (site observations and logs) measures in both intervention (*n* = 4) and usual practice (i.e., control) (*n* = 4) sites (results presented elsewhere). A process evaluation was used to assess implementation. Sites were not randomized but were allocated to receive the intervention based on preference and timing. To ensure that all sites received the benefits of the intervention, the usual practice sites were offered the intervention following the study period. The SOLMo intervention demonstrated promising impact in changing care providers’ attitudes in promoting both PA and HE and in increasing PA among children during the after-school time period. The SOLMo intervention included a needs assessment, tools, workshops, and coaching for care providers to promote HE and PA for children. All sites were expected to meet the two main goals of the intervention (as above), however, the CSH approach allowed for flexibility in how sites individually delivered the intervention. All sites received: daily physical activity bins containing a variety of equipment to facilitate activity in any space, physical literacy playing cards, traditional games playing cards, recipe card lesson plans, and a smoothie kit (blender, measuring cups/spoons, recipe and activity booklets). Additional supportive resources were identified and provided as requested to support sites (e.g., mentorship resources, menu analysis, knife safety). The intervention took place in four after-school sites over a six-month period, September 2016 – February 2017. The purpose of this study was to explore ASC providers’ role and experience promoting HE and PA through SOLMo.

## Methods

Qualitative methods were employed to explore the role of care providers in promoting HE and PA for children attending ASC. Guided by the context-specific nature of this study, focused ethnography was utilized as the guiding method [24].

### Setting: after-school care sites

Each SOLMo ASC site operated independently. Site programming was developed collaboratively among staff members within each site. Care providers were responsible for managing programming, budget, staffing, and licensing/accreditation. Care providers facilitated activities during program hours for children. One of the four sites was located within a school, while the other three had affiliations with local schools. Each received various levels of support from this arrangement (e.g., use of the gymnasium during after-school hours). Three of the four sites were located in an urban centre, with one located rurally. One site was located within a school, another immediately adjacent. The third site was of walkable distance, while the fourth was not walkable for all as the site included a cultural program that drew in students from outside the immediate community. This site also included many newcomers to Canada. Demographic characteristics for children attending the SOLMo sites in this study varied. The age range of children in participating sites was 5–14 years, with a mean age of 8.5 years. Fifty-five percent of participants were girls. Household income data for parents/guardians were collected through a self-reported survey question, “what is your current household income from all sources?” Response options were: less than $25,000; $25, 001-$50,000; $50,001–$75,000; $75,001–$100,000; more than $100,000; don’t know; prefer not to answer. Twenty-eight percent of parents/guardians reported a household income of <$50,000; 30% between $50,000–$100,000; and 42% > $100,001. Site enrollment was provided by site managers and also varied, ranging between 20 and 100 students across sites, with a mean enrollment of 58 students.

### Participants

Through both convenience and purposeful sampling [25], all care providers from the four SOLMo sites (15 in total) were invited to participate in interviews via in-person meetings and through e-mail. A sufficient number of participants (*n* = 13) were recruited to reach saturation [26]. The number of providers per site were as follows: site 1 (*n* = 2), site 2 (*n* = 6), site 3 (*n* = 2), site 4 (*n* = 3). Participants were between 19 and 60 years old, with the mean age of 33 years. Education level was either secondary or postsecondary and work experience ranged from 6 months to 28 years, with a mean of 7 years of experience in the child care industry.

### Data generating strategies

Multiple data generating strategies were utilized in this study to aid in generating a rich understanding of the phenomenon. All strategies were in alignment with focused ethnography [24] and were solely conducted by the first author (PE). At the time of the study, PE was a graduate trainee and registered dietitian with 6 years of practical experience. PE’s exposure to afterschool programs was limited which was advantageous in minimizing research bias (e.g., confirmation bias). PE entered into this study at its initial phase.

Interview data was the predominant source of data. One-on-one semi-structured interviews were conducted and audio recorded. These were conducted over a one-month period following the intervention, at 6-months. The interview guide was developed by the researchers (see supplementary file 1). Questions were specific to care providers’ role in the ASC setting (usual duties and those related to SOLMo), their experiences with SOLMo (resources, personnel), and perceptions of their ability to improve HE and PA opportunities for children (types of activities, barriers, facilitators). Participant observations were completed by the researcher in the field-observer role, and contributed to understanding of the phenomenon [25]. Participant observations included observing care providers facilitating programs at each site. Site visits provided an opportunity to observe how care providers organized programs, and allowed the researcher to ask questions. Site visits were conducted throughout the entire duration of the study (between October 2015 and May 2017, with a minimum of six visits to each site) allowing for prolonged engagement. Field notes summarized each visit which included a reflexive journal entry. The first field note was in mid-October 2015 and last reflexive journal entry was in late-May 2017, after the final interview. Site visits occurred a minimum of three visits to each site during the SOLMo baseline measurement period (October 2015–February 2016) and another minimum of three visits during the SOLMo follow-up period (February–April 2017).

Field visits, field notes, and participant engagement were consistent throughout the two-year duration of the SOLMo intervention. All interview participants reviewed a study information letter and provided written consent in advance of participation. Ethical risks identified were deemed as minimal, relating to the possibility that participants may feel uncomfortable discussing their experiences. As such, participants were advised that they could choose not to answer a question or withdraw from the study without any repercussions.

### Data analysis and rigour

Data analysis was iterative and concurrent throughout data generation. All interviews were transcribed verbatim and organized using the NVivo v11 software program [27]. Latent content analysis was utilized by the first author (PE) for all transcripts and supported the emergence of patterns within the context of the data generated [28].

The criteria of trustworthiness described by Lincoln and Guba was applied [29]. Multiple data generating strategies were triangulated, including interviews and participant observation. Data were integrated through the comparison of interview transcripts, researchers site observation field notes and researcher reflexive journal entries. Coding of transcripts, field notes and journals were first completed manually (i.e., using highlighting and notes in margins), and then transferred to NVivo v11 software program for further coding and development of themes during the analysis phase.

Persistent observation was possible through prolonged engagement and assisted in understanding the unfamiliar setting, site programming/activities, and care provider roles within the ASC setting. Having established relationships prior to interviewing participants contributed to the overall rigour for both data generation and analysis for this study. Reflexivity was considered throughout the study period [30]. Peer (i.e., critical friend) and team debriefings, examination of negative cases, and member checking was completed. An audit trail (i.e., field notes and memoing) was created. Researcher reflexivity [30] and journaling were applied throughout data generation and analysis.

## Results

Thirteen care providers were recruited for interviews. Participants included site leaders (*n* = 5, 38%); full-time staff (*n* = 8, 62%); and part-time staff (*n* = 5, 38%). The majority of the participants were female (*n* = 11, 85%). While diverse, it should be noted that participants were purposefully sampled for the phenomenon of interest. Thus, it is appropriate, and advantageous to examine their responses collectively. Interviews were conducted at each ASC site, lasting between 35 and 90 min.

Data analysis resulted in five major themes related to care providers’ role and experience promoting HE and PA through the SOLMo intervention: (1) enhanced awareness, (2) improved program planning, (3) strong relationships, (4) collaborative approach, and (5) role tension. Overall, interviews with ASC providers and site observations indicated their understanding and support for the use of a comprehensive approach (i.e., CSH) to ensure the well-being and health of children in the ASC setting. We have provided quotes within the text to support our findings. Additional quotes can be found in Table 1.

**Table 1:** Summary of themes with illustrative quotes

Theme 1: Enhanced awareness	
Re-prioritizing HE and PA	… it was School’s Out Let’s Move that got us in the direction of talking about it more openly because I think individually we all thought those things … But we didn’t sit and talk about it and School’s Out got us to the point of sitting and talking about it and, “Okay, so we’re doing this. What else could we offer? What else could we do? … it [SOLMo] made us talk about it more.
Research as an Implementation Tool	… telling them [the children] what it’s [SOLMo] about … Showing them the resources we were given. Just keeping a mindset that is geared towards really emphasizing the physical activity and nutrition part of it.
Theme 2: Improved programming	
Knowledge	I was really honing on the … vigorous. So we started to do a little bit more baseball. Now, baseball is obviously a slow game, but I was changing it up, getting them to do different things with it. So what was happening was I was just taking a regular activity, not reinventing the wheel, just making sure it had a little bit more … vigorous activity
Resources	… incorporating [HE and PA] into our planning more, because now … there’s actually something that we have right there in front of us now instead of thinking, ‘Okay, well, I’m going to look this up. Or I’m going to research it,’ or whatever. No, it’s actually right there. So we can just do it.
Theme 3: Strong relationships	
Unique role as ASC providers	… we benefit because a teacher has a child from September until June and then for lack of better words, they’re done with that child. We get them for years. Years. Like I’ve worked with some of the children since they were 1-years-old to now 9. So, I have years to become that incredibly important person to them … like we have a very special situation.
Role modeling	I think participating in the activities make a difference … If you’re excited about it they’re more likely to be excited about it. If you’re participating and encouraging them to continue trying they’re more likely to … keep trying versus giving up. So, I think that there’s a lot to be done by staff in terms of role modelling to encourage positive choices.
Theme 4: Collaborative approach	
School Partnerships	… we’re lucky in that we’re in this classroom and we’re in this setting, and we have access to whatever it is we need … [I’ve] asked what is it we can take outside as far as equipment, because kids of course want to take things … [and] the school’s really flexible with that.
Community Support	… sometimes we will have a day where it’s two staff, like we’re short-staffed … so our volunteers are very important, especially at the gym, because they’re able to be with those kids who have a little bit more difficult time participating.
Theme 5: Role tension^a^	… people just think all you do is play battleship and you know, that you’re actually overpaid … Because we don’t have all that stuff that we have … to adhere to, with all the measurable outcomes. Well, clearly our job is easier. But you know … This one actually makes it harder … Because it’s like draining. It’s love. And it’s compassion, and it’s all these things. It’s not just doo doo doo … write A, B, C, D … Here’s the answer key and whatever …

^a^No subthemes were identified for this theme

### Enhanced awareness

Interviews revealed the impact SOLMo had on care providers’ ability to promote HE and PA opportunities. Enhanced awareness was reported through participants’ involvement in SOLMo, including meetings with the SOLMo team and supporting child-level data collection. Two subthemes emerged including (1) re-prioritizing HE and PA, and (2) research as an implementation tool. Both subthemes are described below.

#### Re-prioritizing HE and PA

By participating in SOLMo, care providers perceived an enhanced awareness of the importance of providing HE and PA opportunities for children in their care. Care providers described how meetings with the SOLMo team acted as a frequent reminder to prioritize healthy behaviours and encouraged care providers to be “more aware” of healthy eating and physical activity in the ASC. One provider described being: “More conscious about what we were having for snack...And physical activity, too.” Participants reported a general understanding regarding the importance of encouraging healthy behaviours as care providers; however, participants stated these priorities were often previously overlooked during program time due to the hectic nature of the ASC environment.

There was also an increased awareness by *site leaders* in the importance of the care providers’ ability to influence and promote HE and PA for children, which was recognized by *other* care providers as significant. Many felt that through the SOLMo intervention, *site leaders* re-prioritized the need to encourage HE and PA for children, creating awareness for their team as a whole. Enhanced awareness was demonstrated by *site leaders* through team meetings and conversations amongst staff members, which made it easier for *other* ASC providers to also prioritize HE and PA.

#### Research as an implementation tool

ASC providers perceived that the child-level SOLMo data collection (e.g., pedometers, snack observations) enhanced their awareness of SOLMo, including their awareness for promoting HE and PA at their site. Multiple research activities acted as frequent reminders for the project goals (i.e., provide a healthy snack and to increase moderate-to-vigorous PA) and therefore enhanced awareness. For example, pedometers worn by children for data collection served as a reminder for care providers to encourage PA opportunities and often facilitated a conversation with the children regarding what they were for and why they were wearing them. One participant described the benefit of the pedometer as a motivation to participate in physical activity. As they stated: “I thought that the pedometers were great … we did use it [pedometers] like … ‘Well, you want to get more steps, right?’ So and then kids would be more likely to want to participate.” Additionally, not all care providers working within SOLMo sites were full-time. As such, part-time staff reported challenges in communication of new programs or initiatives. Research activities such as child-level baseline data collection (e.g., pedometers, snack observation) served as a means to signal to all staff that the SOLMo intervention was taking place.

### Improved program planning

While programs e shared a common goal of ensuring the well-being of children as a priority. Two subthemes of (1) knowledge and (2) resources emerged. An improvement in program planning processes to support HE and PA through the knowledge gained and resources provided by SOLMo was reported by participants. Efforts required to plan activities that were of interest to children, combined with the chaotic environment, made planning for HE and PA opportunities difficult at times. Subthemes are described in detail below.

#### Knowledge

Participants spoke of an increase in knowledge gained in the areas of HE and PA from participating in SOLMo and discussed how this knowledge allowed them to intentionally plan activities which regularly included HE and PA opportunities. Care providers mentioned program planning as a significant component of their role. The program planning process for each site varied (i.e., some daily, weekly, or monthly), however, program planning was important as it provided a loose structure for each site’s daily operations. A participant described how even though their organization encourages PA, she learned of MVPA recommendations for children due to being a part of SOLMo: “I would say what has changed is just my perspective. I did know that it’s important, but I didn’t think every day had to incorporate at least 60 minutes of exercising or of [moderate-to-vigorous] physical activity.”

#### Resources

Care providers recognized the physical environment impacted their ability to promote healthy lifestyle behaviours. Participants indicated opportunities to promote HE and PA were challenging given their limited access to resources, and reported how SOLMo resources (i.e., ideas, supplies) allowed them to make changes to the environment to improve HE and PA opportunities. This was particularly relevant when providing PA opportunities within small spaces, as one participant illustrated: “that [daily physical activity] bin in particular allows us to do things in a smaller space, which is really useful.”

### Strong relationships

Care providers identified themselves as being effective health promoters. Care providers perceived they had a significant ability to impact children’s health behaviours (i.e., HE and PA) through the strong relationships they had with children, which were established over time. The importance of building a relationship with each child was reported as a main priority for care providers; participants spoke of how the relationship with each child needed to be established before promoting health. Trust and established relationships with the children were discussed as crucial components of their role. By creating a positive social environment, open communication, and conversations between the care provider and children, promoting HE and PA was made easier. Two subthemes emerged within the context of ‘Strong Relationships’: (1) unique role as ASC providers and (2) role modeling, as described in detail below.

#### Unique role as ASC providers

Care providers perceived their strong relationship with children was established through their unique role as ASC providers. Care providers interact daily with children, and sometimes these interactions occur over long a period of time, lasting years for some children. This continued interaction in a less structured environment, combined with an emphasis on establishing connection, fostered an important bond between the care provider and children.

Once a relationship was formed with a child and trust was established, care providers perceived this bond as advantageous in their ability to influence children’s health behaviours. While hectic at times, the less structured nature of ASC programs allowed for meaningful connections between care providers and children. Forming relationships, building trust, and creating mutual respect with children supported care providers in their ability to promote HE and PA because “once that respect is established and that relationship is built … it makes a difference … they’re super respectful and wanting to be here. And so I feel like what I [the care provider] say to them matters.” The relationship care providers developed with children was reported as unique to their role as ASC providers, and enhanced their ability to influence and promote health behaviours.

#### Role modeling

Many care providers recognized the importance of their indirect influence through role modeling of HE and PA. Participants commented on the responsibility of care providers to exhibit health behaviours and the importance of ‘practicing what you preach’ by role modeling healthy behaviours to encourage and positively influence children’s behaviours. Participants recognized their ability to influence children through their own thoughts and actions, and could bring energy and excitement to activities. The need to be conscious of their behaviours exhibited during program time was described by one participant: “We are really big influencers on them... we’re role models.” Participants described modeling healthy behaviours by participating in the activities with the children (e.g., playing games, sitting with children and eating the healthy snacks prepared) as well as being conscientious about not bringing in unhealthy food choices. Activities were not mandatory in the ASC setting due to the nature of child-led programming, and role modeling by care providers was reported to improve children’s participation, especially those children who were less likely to participate. While some challenges in participating in activities were voiced due to the need to oversee the entire program or other activities, care providers agreed that their engagement improved participation levels.

### Collaborative approach

Care providers discussed the importance of a collaborative approach in their ability to successfully promote HE and PA within the ASC setting, including the significance of connections formed within their community. Partnerships with schools were described as impactful; however, participants indicated challenges were often encountered when establishing these relationships. Once a partnership was established, participants reported a reliance on schools. Many sites had limited resources, thus care providers indicated a collaborative approach was required to improve HE and PA opportunities. Two subthemes were revealed: (1) school partnerships and (2) community support, and are described below.

#### School partnerships

Three of the four SOLMo sites were within walking distance to schools, one within the school itself. For some sites, the relationship with the school and school staff was significant and positive and supported HE and PA opportunities. This included opportunities to access school resources (e.g., equipment, space) during program hours. Unfortunately, this ‘fundamental’ support between the school and ASC site was not observed by all, and was recognized as atypical by most. Throughout the SOLMo intervention, conversations with care providers revealed challenges in establishing these relationships.

In comparison to ASC sites, schools are generally better equipped with resources. Participants agreed that support from school improved care providers’ ability to promote HE and PA. Participants from sites with established partnerships spoke of their reliance on the school for resources including access to school gymnasium, sports equipment, outdoor play space, kitchen space, and food donations which often came as leftovers from school events. While not all sites had strong relationships with the local school, ASC providers perceived access to shared school resources would improve their ability to promote HE and PA.

#### Community support

Care providers also spoke of the connections made with the community as necessary in promoting HE and PA. This was especially true in the context of the financial constraints faced for sites offering a service-fee free program. Due to budgetary challenges, community support for food donations was essential, as described by one participant: “… we have limited resources as far as our budget’s set out for the year. And we do rely on the food bank. And that dictates basically what it is we can offer.” Community partnerships were also invaluable in improving PA opportunities for ASC sites. Access to resources in the community was described as significant in the care provider’s ability to plan PA opportunities without adding additional costs. Connecting with other sites within the ASC community was viewed as an opportunity to improve HE and PA opportunities through idea sharing. Support from families was also regarded as beneficial. Conversations initiated with parents on HE and PA encouraged parental involvement and support for SOLMo. Community volunteers were also viewed as essential, especially during times of high staff turnover, supporting care providers by leading and facilitating activities during program time. The ‘extra bodies’ assisted care providers in improving HE and PA promotion by engaging with children to encourage participation.

### Role tension

The role of an ASC provider is unique. They provide overall care and support the educational needs for children. Throughout the SOLMo intervention, the responsibilities of the care provider as being both a child minder and educator was observed, and challenges of this dual role were revealed. These challenges extended to how care providers were able to support HE and PA opportunities for children. While care providers embraced their dual role, they experienced an internal tension, describing this tension related to the dynamic day-to-day operations, less structured nature of the ASC setting, and the perceived lack of support within their community. Care providers recognized their ability to influence children’s health behaviours and aimed to plan programs that incorporated HE and PA opportunities. However, observations and discussions with participants exposed the reality of the care provider’s role, which predominantly prioritized safety (i.e., managing and ensuring safety of children), and left little time to facilitate planned health promoting activities during ASC hours. Additionally, handling behavioural issues among the children was reported as a frequent deterrence.

Participants spoke of the role tension experienced within the school community. Care providers shared their perceptions of feeling undervalued by members of the school, notably teachers. Comparisons between the role of ASC providers and teachers emerged at various points throughout the SOLMo intervention. A perceived hierarchy within the school community was described, with some participants perceiving their role as inferior to other staff members within the school. A lack of respect was perceived compared to teachers in the school setting. Participants perceived their unique role, the dual role of both a child minder and educator, was unrecognized by most.

The perceived low regard for the ASC provider role came from the school community, families, and the general public. This perceived lack of a sense of community or belonging, and value and professionalism in their role affected the quality of care provided by care providers. The negative perception was challenging for care providers to feel confident and competent in their role, impacting their ability to provide quality care for children, including their ability to promote health. The lack of understanding for their role was described by one participant in the following: “… the perception that we’re just child care workers sometimes lays on people pretty hard … sometimes that actually weighs on staff. ‘Well why am I doing it? The family just views me as a babysitter.”

## Discussion

Emerging research suggests a comprehensive approach is needed to address the multiple environmental factors influencing children’s health behaviours. As such, taking a CSH approach in the ASC setting is warranted. While it was anticipated that the ASC provider plays a crucial role in providing HE and PA opportunities in the ASC setting, limited research was available regarding their role specifically. The goal of this research was to explore the role of the care provider and their perceived ability to promote HE and PA opportunities within ASC sites participating in the SOLMo intervention. Five themes resulted from this study, revealing care providers’ perceptions and experiences of promoting HE and PA through the SOLMo intervention. Generally, a positive experience was reported by participants.

Participants reported an improved awareness of HE and PA as a result of the intervention. An enhanced awareness was also seen in the after-school intervention reported by Dobson et al. [21]. The authors concluded that care providers showed increases in knowledge and confidence levels in offering HE and PA opportunities. Through focus groups, ASC providers participating in the intervention reported “feeling empowered and excited” in their role as ASC providers as a result of the intervention (Dobson et al., [21], p. 14,). The enhanced awareness in the present study was reported through the re-prioritization of HE and PA, with site leaders as key individuals leading the change within sites. The significant role of the site leader in implementing health interventions for children has also been reported by other studies [31, 32]. Beets et al. [33] found changes made to improve PA opportunities at ASC sites were primarily driven by the ASC leader, despite a lack of formal or routine changes to site programs observed at post-intervention. Similarly, as the leader of a school, principals are viewed as essential to successfully implementing school-based interventions [32].

Although ASC sites are non-curriculum based, program planning is an important component. Participants reported improvements to program planning as a result of the SOLMo intervention. While care providers acknowledged a general understanding of HE and PA recommendations, an increase in their knowledge was reported. Care providers receive little education on HE and PA during their formal training, and thus SOLMo served as a means to provide professional development in health behaviours. This has been similarly reported in other studies with this population [34]. Studies on HE and PA interventions implemented in schools and after-school child care centres suggest teachers, care providers, or the persons implementing the program need ongoing training and support in order to feel confident and competent in their ability to implement changes [35–37]. Care providers, much like teachers and staff within the school setting, have a significant impact on the healthy choices of children in their care. During formal training for ASC providers (e.g., early learning and child care certification), it would be beneficial to integrate course material which addresses HE and PA in the ASC setting. Providing instruction, resources, and opportunities to understand HE and PA recommendations for children and youth can better equip ASC providers with knowledge and skills to foster healthy habits among students. Furthermore, investment by ASC organizations and supportive granting for regular professional development opportunities would allow care providers to improve their ability promote HE and PA.

A common challenge ASC programs experience is low attendance and participation [38]. As a strategy, care providers use child-led programming to improve participation and thus aimed to change activities frequently to tailor to childrens’ interests. Limited access to equipment and a limited budget made planning for PA or HE programs challenging for SOLMo care providers and is a challenge ASC programs regularly face [18, 39]. Additional resources helped to improve healthy opportunities for SOLMo sites, and has also been similarly reported in other after-school interventions [39, 40]. Huberty et al. [40] found an increase in MVPA participation for both boys and girls with the presence of equipment (e.g., balls, jump-ropes, hula-hoop). The increase in variety of activities, with the addition of resources from an intervention, helps to increase interest and participation in PA among children in the ASC setting [20, 40].

Care providers’ influence on children’s HE and PA behaviours as a result of the strong relationships they had formed with the children was revealed. The unique bond ASC providers share with children built trust and encouraged open communication. This unique bond was similarly identified by Leos-Urbel [38] who described how the interactions between care providers and children were associated with higher reading scores and academic success within an ASC setting. In addition to strong relationships, role modeling was also viewed as imperative in the present study. Role modeling of positive health behaviours, such as participating and actively engaging with children during activities, was recognized as a significant component of the care provider role within the ASC setting. The effects of role modeling to influence children’s health behaviours have been reported in other ASC settings [40], and were also found in other contexts, such as teachers within a school [41–44], and mentors within the community [45, 46]. Additionally, He and colleagues [42] demonstrated the need for teachers to have healthy attitudes and be positive role models because of the considerable time they spend with children. Similar to the findings of the present study, Zarrett et al. [39] reported on the impact of care provider’s facilitation style; the importance of care providers actively engaging with children to improve PA participation through verbal encouragement and participation in the activity.

The ability to collaborate with the school and community was perceived as essential. Care providers viewed partnerships with the school and community as crucial in their ability to access resources they would not have otherwise had. The collaborative approach required is consistent with findings in the literature, including after-school [47, 48], community [49], and school settings [45]. The conflicts with the school community identified by ASC providers in this study were unexpected. Participants revealed the challenges care providers experienced in their efforts to work collaboratively with schools. To our knowledge, the relational conflicts ASC providers experience with schools or partnerships within the community has not been investigated. This may be due to the limited research on the care providers’ role in promoting HE and PA within the ASC setting. These findings underscore the need for coordination between the ASC, school, and community settings to collectively support the health and wellbeing of students. School communities should consider how they can support ASC providers through partnerships and connections with programs in their community (both co-located and offsite). Additionally, higher-level support through formal joint-use agreements for infrastructure and equipment (e.g., between school boards, municipal bodies, and ASC centres) would help support coordination of efforts to improve PA and HE in the in after-school hours.

Role tension was commonly experienced by care providers in this study. Their unique role as both care provider and educator in the ASC setting was embraced by participants, however, challenging at times. There are multiple responsibilities faced by ASC providers which have been similarly described as work-related stress by child care workers in daycare centres [50]. The challenges of the ASC providers’ dual role of providing overall care and educating school-age children combined with the hectic nature of the ASC environment was perceived to create this role tension. Furthermore, the perceived lack of support, in particular the relationship with the school, was a trigger for the tension experienced. A perceived lack of respect by the community was also mentioned. The misconception of their role as ‘babysitter’ by parents or the general public was found to be discouraging. Participants indicated this misconception affected their ability to feel a sense of pride in their role and to provide quality programming and care. Ongoing communication between ASC sites, schools, and families regarding the role of the care provider may mitigate these challenges. Further, it is possible that the role tension experienced by care providers may become less pronounced as health promotion strategies in the ASC setting become more widespread.

### Strengths and limitations

Strengths of this research include the consistent and prolonged engagement with participants. Field visits by the primary researcher over the two-year period of the SOLMo project provided background knowledge regarding the after-school setting and allowed for the development of strong relationships with participants [25, 28]. Additionally, there was a diverse range of participants in: age, child care experience, full-time, part-time staff, and included site leaders in addition to front-line staff. While this intervention took a CSH approach, it is known that comprehensive approaches require time to implement [51]. Further time implementing the intervention may have yielded additional insights from care providers that were not captured in the current study. Furthermore, the primary researcher was also the project coordinator for the SOLMo intervention, which had the potential to create a social desirability bias whereby participants underreported socially undesirable attitudes or behaviours. The researcher was mindful in establishing a relationship with participants to gain trust prior to conducting interviews to encourage participants to speak freely. To enhance the quality and transparency of all aspects of the research, we applied the 21-item Standards for Reporting Qualitative Research checklist [52], see supplementary file 2.

## Conclusions

Childhood obesity remains a major public health concern in Canada, and comprehensive strategies which focus on prevention are needed. The ASC setting is a logical and ideal setting to promote health of children and youth. The critical hours of 3:00–6:00 p.m. are gaining interest as a ‘window of opportunity’ to promote healthy lifestyle behaviours among school-aged children. By adopting the CSH framework, the SOLMo after-school intervention used a whole-setting approach to promote and improve HE and PA opportunities. Recognized as critical influencers, the care providers’ role and their experiences in participating in SOLMo was explored in this study. Findings of this study highlight the role of care providers and contribute to the literature on comprehensive approaches to health promotion within ASC settings. Our results contribute to the implementation knowledge base. Specifically, this study highlights the opportunity and need to integrate HE and PA in after-school care training (both pre-service and ongoing professional development) to better equip care providers for a role that includes and prioritizes child health and wellbeing. Support at the site-level, through guidelines and resources and formalized policy for HE and PA can serve to further integrate health promotion in the ASC setting. In addition, it is recommended that where possible, regional, and local jurisdictions can support the coordination between schools, ASC programs and the community for the benefit of children and staff. These results will help to improve best practice guidelines to support care providers in promoting healthy lifestyle behaviours for school-aged children in ASC settings.

## Supplementary information

**Additional file 1 Supplementary file 1**: Interview Guide. Study interview guide developed by the researchers for interview with after-school care providers.

**Additional file 2 Supplementary file 2**: Standards for Reporting Qualitative Research Checklist. SRQR Checklist completed for this qualitative study.

## Data Availability

The data used in the current study is available from the corresponding author on reasonable request and conditional HREB approval.

## Abbreviations

ASC: After-school care
CSH: Comprehensive school health
HE: Healthy eating
MVPA: Moderate to vigorous physical activity
PA: Physical activity
SOLMo: School’s out … let’s move
