# Supplementary material for: Canadian after-school care providers’ perceived role promoting healthy lifestyles: a focused ethnography
Source: BMC Public Health. 2020 Aug 25;20:1279. doi: 10.1186/s12889-020-09369-y (PMC7445905; doi:10.1186/s12889-020-09369-y)
Supplement: Supplementary file 1 — Additional file 1 Supplementary file 1: Interview Guide. Study interview guide developed by the researchers for interview with after-school care providers. [file 12889_2020_9369_MOESM1_ESM.docx]

**Background Information:**

- *Thank them first. Ensure read and reviewed information letter and have written consent (double check before interview)*
- *Reiterate that this is confidential – data only shared with research team (Dr. Storey’s team) – not shared with site managers/supervisors. NEVER will identify your site individually.*
- *Discuss purpose of the interview*
  - *Experiences of* ***their role*** *in implementing the School’s Out…Let’s Move project and to understand how* ***SOLMo affected their ability to facilitate healthy eating and physical activity opportunities*** *for children in the after-school care setting*
- *Logistics of the interview*
  - *Recorded – ask if they are okay being recorded; let them know when you are turning on and off*
  - *How long it will take*
  - *Informal conversation – feel free to go back to anything*
  - *May feel that some questions are quite similar*
- *Do you have any questions before we begin?*

**Interview Guide**

| Site Name |  |
| --- | --- |
| Age range of kids at site (e.g., K-6)  Number of students registered at site |  |
| How long have you been at this site? |  |
| Have long have you been working as a care provider in an after-school care setting? |  |
| Are there any other programs (besides SOLMo) being run in your after-school site at this time (i.e. Leader In Me)? |  |
| **Demographics for Publication:** |  |
| What is your level of education? What degree(s) do you have? |  |
| Male / Female |  |

1. To start, would you be able to describe your role at the after-school care site here at *(site name)*?
   1. Are you here at the program every day?
2. **Can you tell me what makes it easy for you to offer opportunities for healthy eating and physical activity for the kids at your site?**
   1. What makes your role as a care provider fun?
3. **Can you tell me what it makes it hard for you to offer opportunities for healthy eating and physical activity for the kids at your site?**
   1. What makes your role difficult as a care provider?
4. **Can you tell me about your experience with the School’s Out...Let’s Move project that was implemented at your site this past year?**

*Probing Qs:*

- 1. Were you aware of the implementation of the project?
  2. Were you aware of the resources, such as the DPA bin, PA recipe cards, AHS Healthy Eating Manual or Healthy Smoothie Blender Package, provided for the project?
  3. Did you have any opportunities to use the resources provided?

1. **Did your experience of the SOLMo project lead to any changes in how you ran the program at your site?**

*Probing Qs:*

1. For PA and/or HE opportunities…In what way? Are you able to provide some examples?

(facilitation style? Eg. leading, participating, supervising)

1. **What are some key factors that have helped you in implementing the School’s Out…Let’s Move project?**
   1. Probes: resources, meetings with SOLMo team, people (relationships)
   2. Was your site and staff supportive of the project? What does this “support” look like?
      1. Probe: from the beginning, after some time, etc.?
2. **What are some barriers that have hindered you in implementing the School’s Out…Let’s Move project?**
3. *Responses could include: resistance with parents/staff, building trust with key stakeholders (including parents), staff turnover, buy-in, etc.*
4. **If you had to give advice to a care provider new to implementing the School’s Out…Let’s Move project, what would it be?**
   1. Or advice on how to improve in promoting HE and PA to children at your site
5. Is there anything you would like to add?

*Conclude interview by thanking them again for their time and indicate that once the findings from this research have been analyzed, reports will be generated and distributed to different audiences (e.g., school administrators).*
