# Supplementary material for: Canadian after-school care providers’ perceived role promoting healthy lifestyles: a focused ethnography
Source: BMC Public Health. 2020 Aug 25;20:1279. doi: 10.1186/s12889-020-09369-y (PMC7445905; doi:10.1186/s12889-020-09369-y)
Supplement: Supplementary file 2 — Additional file 2 Supplementary file 2: Standards for Reporting Qualitative Research Checklist. SRQR Checklist completed for this qualitative study. [file 12889_2020_9369_MOESM2_ESM.docx]

Standards for Reporting Qualitative Research Checklist: After-school care providers perceived role

| S1 | Title | Canadian after-school care providers’ perceived role promoting healthy lifestyles: A focused ethnography |
| --- | --- | --- |
| S2 | Abstract | After-school care (ASC) programs have garnered interest in recent years as the hours of 3:00–6:00p.m. are an opportune time for children to engage in healthy behaviours, specifically healthy eating (HE) and physical activity (PA). Care providers are major influencers within the ASC setting, impacting HE and PA opportunities for children. However, little is known regarding the role care providers play in health promotion interventions in the ASC setting, specifically those using comprehensive approaches. The purpose of this research was to explore care providers’ perceptions and experiences implementing the ASC health promotion intervention School’s Out…Let’s Move (SOLMo). SOLMo was guided by the evidence-based comprehensive school health (CSH) framework, and included a focus on HE and PA. This research was guided by the qualitative method focused ethnography. Semi-structured interviews with care providers (n=13) taking part in SOLMo were conducted to explore their perceptions of the intervention. Participant observation was included as part of data generation to further understand care providers’ roles. Through latent content analysis, five themes emerged: 1) enhanced awareness; 2) improved programming; 3) strong relationships; 4) collaborative approach; and 5) role tension. As major influencers, care providers’ play a crucial role, and these results will contribute to implementation strategies used to promote healthy lifestyle behaviours for children. |
| S3 | Problem formulation | Care providers are major influencers within the ASC setting, impacting HE and PA opportunities for children. However, little is known regarding the role care providers play in health promotion interventions in the ASC setting, specifically those using comprehensive approaches. |
| S4 | Purpose or research question | The purpose of this research was to explore care providers’ perceptions and experiences implementing the ASC health promotion intervention School’s Out…Let’s Move. |
| S5 | Qualitative approach and research paradigm | The context-specific nature of this study led to focused ethnography being the best method suited to inform the purpose of this study. The researcher’s theoretical position for this study includes a constructivist perspective; comprised of the relativist ontology, the belief in multiple realities, and a subjectivist epistemology, to mean for the co-creation of data generation by researchers and participants. Together these perspectives create the foundation of a constructivist perspective, and informed the research process in its entirety. |
| S6 | Researcher characteristics and reflexivity | The primary researcher has a background and food and nutrition as a practicing dietitian, and is also a parent and thus has knowledge regarding the promotion of healthy eating and physical activity. The primary researcher also had some knowledge with regards to the child care setting as a participating parent with assumptions based on this minimal knowledge/participation. The primary research was not familiar with the specific after-school care setting studied in this research. The primary researcher used journaling, completed throughout data collection and analysis after each data collection opportunity and/or field visit, to allow for reflection at an in-depth level to experience and interpret the data. Journaling was also important for the researcher to maintain self-awareness in their role as a researcher and outsider, while taking into consideration the views of the participants. |
| S7 | Context | All interviews, meetings and researcher-participant interaction where done on site at ASC sites to minimize time and work day disruption for participants and allowed increased participant observation by researchers. E-mail and/or phone was used throughout study to ensure good communication and facilitate a strong relationship between researchers and participants. |
| S8 | Sampling strategy | Convenience and purposeful sampling |
| S9 | Ethical issues pertaining to human subjects | This research received ethics approval from the University of Alberta Human Research Ethics Board, under the project name “Evaluating the impact of School’s Out…Let’s Move after-school program on children’s health and health equity” No. Pro00058006. Written and verbal consent was gathered from all participants in the study. |
| S10 | Data collection methods | One-on-one semi-structured interviews*,* which prompted relevant questions while allowing for additional questions to emerge to elicit detail and in-depth descriptions from participants’ experiences, were conducted and audio recorded.  Participant observations were completed by the researcher in the field-observer role, and contributed to understanding the role of ASC staff by providing direct observation of care providers’ ability to facilitate HE and PA activities within the ASC setting. Field work allowed for informal interactions and conversations with care providers and others (e.g., volunteers, school principal, and teachers) and provided the researcher with insight regarding the daily routines and general practices involving HE and PA promotion within the ASC setting. Extensive field notes were taken throughout each site visit over the SOLMo study period.  Reflective journaling followed each site visit or meeting with participants to document the researcher’s thoughts, feelings, interpretations or reflections on what was observed or discussed. |
| S11 | Data collection instruments and technologies | Field work and participant observation assisted in the development of the interview guide. Questions were specific to care providers’ experiences with the SOLMo intervention, and their perceptions of their ability to improve HE and PA opportunities for children in the after-school setting. Initial interviews informed subsequent interviews with adjustments and revisions made based on participant responses. Member checking was part of the interview guide, completed at the end of interviews, in the form of summary questions. All interviews were audio recorded and transcribed verbatim. |
| S12 | Units of study | Thirteen care providers were recruited for interviews. Participants included site leaders (n=5); full-time staff (n=8); and part-time staff (n=5). The majority of the participants were female (n=11). Participants were between 19 and 60 years old, with either secondary or postsecondary education. Work experience ranged from 6 months to 28 years in the child care industry. Interviews were conducted at each ASC site, lasting between 35-90 minutes. |
| S13 | Data processing | Interviews transcribed verbatim and organized using NVivo v11 software program |
| S14 | Data analysis | Data analysis was iterative and concurrent throughout data generation process. Field work and participant observation assisted in the development of the interview guide; initial interviews informed subsequent interviews with adjustments and revisions made based on participant responses. Upon completion of the transcription of interviews, transcripts were read multiple times prior to the coding process to ensure accuracy and assist the researcher in immersion with the data. Latent content analysis was utilized to identify, code, categorize, and ultimately develop themes. |
| S15 | Techniques to enhance trustworthiness | Audit trail (i.e., field notes and memoing) prolonged engagement (participant observation), peer (i.e., critical friend) and team debriefings, examination of negative cases, and member checking. |
| S16 | Synthesis and interpretation | Data analysis resulted in five major themes: (1) enhanced awareness, (2) improved program planning, (3) strong relationships, (4) collaborative approach, and (5) role tension. Overall, interviews with ASC providers and site observations indicated their understanding and support for the use of a comprehensive approach (i.e., CSH) to ensure the well-being and health of children in the ASC setting. |
| S17 | Links to empirical data | Quotes from interviews are used as evidence. Field notes and researcher journals also provided evidence in the development of themes. |
| S18 | Integration with prior work, implications, transferability, and contribution(s) to the field | Emerging research suggests a comprehensive approach is needed to address the multiple environmental factors influencing children’s health behaviours. As such, taking a CSH approach in the ASC setting is warranted. While it was anticipated that the ASC provider plays a crucial role in providing HE and PA opportunities in the ASC setting, limited research was available regarding their role specifically. The goal of this research was to explore the role of the care provider and their perceived ability to promote HE and PA opportunities within ASC sites participating in the SOLMo intervention. Five themes resulted from this study, revealing care providers’ perceptions and experiences of the SOLMo intervention. Findings of this study highlight the role of care providers and contribute to the literature on comprehensive approaches to health promotion within ASC settings. Results will improve best practice guidelines to support care providers in promoting healthy lifestyle behaviours for school-aged children in ASC settings. |
| S18 | Limitations | The SOLMo intervention was delayed due to recruitment challenges and thus the intervention period was reduced from one year to six months. The CSH framework is complex and challenging to fully implement and require time to implement. A longer study period may have had an impact to change the experiences and perceptions care providers reported in this study. The primary researcher was also the project coordinator for the SOLMo project, which had the potential to create a social desirability bias by participants. The topic of this research, however, was not sensitive. The researcher was mindful in establishing a relationship with participants to gain trust prior to conducting interviews to encourage participants to speak freely. Moreover, social desirability bias has been reported as rare in qualitative interviews within a similar culture |
| S20 | Conflicts of interest | The authors declare that they have no competing interests |
| S21 | Funding | This work was supported by the generous support of the Stollery Children’s Hospital Foundation through the Women and Children’s Health Research Institute (WCHRI) [Innovation grant number RES0026796]. |
